# What does LTP tell us about the roles of CaMKII and PKMζ in memory?

**DOI:** 10.1186/s13041-018-0420-5

**Published:** 2018-12-28

**Authors:** Todd Charlton Sacktor, André Antonio Fenton

**Affiliations:** 1 Department of Physiology and Pharmacology, The Robert F. Furchgott Center for Neural and Behavioral Science, State University of New York Downstate Medical Center, Brooklyn, NY 11203 USA; 2 Departments of Neurology and Anesthesiology, State University of New York Downstate Medical Center, Brooklyn, NY 11203 USA; 3 Center for Neural Science, New York University, New York, USA; 4 Neuroscience Institute at the NYU Langone Medical Center, New York, USA

## Abstract

In “Criteria for identifying the molecular basis of the engram (CaMKII, PKMζ),” Lisman proposes that elucidating the mechanism of LTP maintenance is key to understanding memory storage. He suggests three criteria for a maintenance mechanism to evaluate data on CaMKII and PKMζ as memory storage molecules: *necessity*, *occlusion*, and *erasure*. Here we show that when the criteria are tested, the results reveal important differences between the molecules. Inhibiting PKMζ reverses established, protein synthesis-dependent late-LTP, without affecting early-LTP or baseline synaptic transmission. In contrast, blocking CaMKII has two effects: 1) inhibiting CaMKII activity blocks LTP induction but not maintenance, and 2) disrupting CaMKII interactions with NMDARs in the postsynaptic density (PSD) depresses both early-LTP and basal synaptic transmission equivalently. To identify a maintenance mechanism, we propose a fourth criterion — *persistence.* PKMζ increases for hours during LTP maintenance in hippocampal slices, and for over a month in specific brain regions during long-term memory storage in conditioned animals. In contrast, increased CaMKII activity lasts only minutes following LTP induction, and CaMKII translocation to the PSD in late-LTP or memory has not been reported. Lastly, do the PKMζ and CaMKII models integrate the many other signaling molecules important for LTP? Activity-dependent PKMζ synthesis is regulated by many of the signaling molecules that induce LTP, including CaMKII, providing a plausible mechanism for new gene expression in the persistent phosphorylation by PKMζ maintaining late-LTP and memory. In contrast, CaMKII autophosphorylation and translocation do not appear to require new protein synthesis. Therefore, the cumulative evidence supports a core role for PKMζ in late-LTP and long-term memory maintenance, and separate roles for CaMKII in LTP induction and for the maintenance of postsynaptic structure and synaptic transmission in a mechanism distinct from late-LTP.

## Introduction

For many years the notion of a persistently active, long-term memory storage molecule seemed superfluous. Active enzymatic processes were thought to maintain only short-term memory, not long-term memory. This idea was consistent with the view that most molecular signaling events in cells were short-lived. As shown in model systems such as *Aplysia californica*, experiences that produced short-term memory induced strong synaptic stimulation that increased the amounts of short-lived second messengers within neurons to transiently activate protein kinases [1]. The transient activation of these kinases briefly enhanced synaptic strength to modify the neuronal circuits underlying behavior for a short time. If the stimulation induced by experience was strong enough, some of these signaling molecules would also transiently upregulate gene expression, leading to a brief period of new synthesis of proteins that could support synaptic growth within the circuits [2]. Once formed, these new synapses were presumed to be stable anatomical structures that would permanently alter the circuits to maintain long-term memory without the requirement for special persistently active memory molecules.

In the mid-1980’s, however, three highly creative thinkers, now sadly all departed: Francis Crick, James H. Schwartz, and John Lisman, championed the notion that memory might be maintained by active mechanisms, in particular persistently active protein kinases. All three suggested the possibility that with strong stimulation the kinases that mediate short-term memory could be converted from transiently active, second messenger-dependent forms into persistently active, second messenger-independent forms. These autonomously active enzymes, “cognitive kinases” in Schwartz’s phrase [3], might then sustain enhanced synaptic transmission during persistent forms of memory. Crick proposed a theoretical model involving positive feedback between molecular dimers, analogous to the mechanism for stability of the double helix of DNA [4]. Schwartz focused on the persistent action of the cAMP-dependent protein kinase (PKA), which mediated short-term memory in *Aplysia*, through the degradation of its autoinhibitory regulatory unit. He proposed that this persistence underlies an intermediate-phase of memory between short- and long-term memory [5].

Lisman’s proposal focused on Ca^2+^/calmodulin (CaM)-dependent protein kinase II (CaMKII), an abundant protein of the PSD of glutamatergic synapses, that built on the earlier biochemical work on CaMKII by Schwartz [6] and Mary Kennedy [7]. Schwartz and Kennedy had found that, once CaMKII was activated, its autophosphorylation reduced the enzyme’s requirement for Ca^2+^/CaM, converting the kinase into an autonomously active form. Lisman championed the idea that the autophosphorylation of CaMKII on threonine-286 (T286) and the subsequent autonomous activity of the enzyme described by Kennedy maintained LTP, a leading putative physiological substrate of memory in vertebrates [8, 9].

In the early 1990’s, a third kinase, protein kinase C (PKC), was also found to have an autonomously active form. By this time, PKC had been shown not to be the product of a single gene, but a small gene family of ~ 11 isoforms, divided into conventional, novel, and atypical classes [10]. Most of the PKCs were activated by second messengers that bound to the kinase’s regulatory domain, releasing the domain’s autoinhibition of the PKC catalytic domain. One of the atypical isoforms, however, termed PKMζ, was an independent PKC catalytic domain that lacked an autoinhibitory regulatory domain, rendering it an autonomously active kinase [11]. Unlike the other isoforms that were activated only briefly in LTP by transient increases in second messengers such as Ca^2+^ and diacylglycerol, PKMζ persistently increased in LTP maintenance through a protein synthesis-dependent mechanism. PKMζ was formed in neurons from a dedicated dendritic PKMζ mRNA, which normally was translationally repressed and unable to synthesize protein [11–14]. Strong synaptic activity, however, derepressed the PKMζ mRNA, driving de novo synthesis of the autonomously active kinase. Over the years, as interest in LTP as a potential mechanism of memory expanded rapidly, evidence for the roles of both CaMKII and PKMζ in these processes grew [15–18].

## Inclusion vs. exclusion of evidence for CaMKII and PKMζ

In his review, Lisman compares the evidence supporting CaMKII and PKMζ in LTP and memory maintenance, focusing on three criteria that he proposes to identify a maintenance mechanism: *necessity*, *occlusion*, and *erasure* [16]. He concludes that there are data supporting both kinases, but the evidence for CaMKII is stronger. Lisman’s conclusion, however, requires excluding evidence in support of PKMζ from a large number of studies that use the peptide PKMζ-inhibitor ZIP, which mimics the pseudosubstrate inhibition of the PKCζ regulatory domain that is missing from PKMζ. These data were excluded because of ZIP’s potential off-target effects [19–22], while ignoring the direct electrophysiological evidence to the contrary in the excluded LTP and memory experiments [23–25].

To reach his conclusion that ZIP’s effects are non-specific, Lisman excluded three sets of data. First, in normal animals, ZIP’s ability to reverse LTP and erase long-term memory crucially depends upon the peptide’s ability to block the specific mechanism of action by which PKMζ potentiates postsynaptic AMPAR responses [26–28]. PKMζ causes and maintains potentiation of postsynaptic AMPAR responses by a mechanism distinct from CaMKII or other PKCs. Instead of increases in AMPAR unit conductance or exocytosis of the receptor to the plasma membrane, PKMζ maintains synaptic potentiation by decreasing postsynaptic AMPAR endocytosis [26, 29]. This action of PKMζ, which causes and sustains a doubling of the number of functional AMPAR channels at postsynaptic sites [30], is mediated specifically by inhibiting GluA2 subunit-dependent endocytosis [29]. If this GluA2-dependent endocytosis is blocked by the peptide GluR23Y, ZIP has no effect on LTP or long-term memory [26–28]. These results demonstrate that ZIP targets PKMζ’s mechanism of action and thus reverses LTP maintenance and erases memory by acting on PKMζ or on a molecule with a very similar action.

Second, ZIP’s effects in mutant PKMζ-knock-out mice on LTP and memory [19, 20] are not due to non-specific effects of the drug, but rather the recruitment in the mutant mice of a different target of drug action than in wild-type mice [25]. Antisense oligodeoxynucleotides that specifically block new synthesis of PKMζ prevent the formation of late-LTP and long-term memory in wild-type mice, but not in PKMζ-knock-out mice, demonstrating that the maintenance mechanisms in these two genotypes are different. In mice lacking PKMζ, another atypical PKC isoform, PKCι/λ, which like PKMζ is sensitive to ZIP, becomes persistently active in late-LTP and long-term memory to compensate for PKMζ.

Third, bath applications of ZIP block the synaptic potentiation caused by postsynaptic perfusion of PKMζ or PKCι/λ, but not the potentiation induced by phorbol esters, activators of the full-length conventional and novel PKCs, demonstrating selectivity of the agent’s action within neurons [25]. Moreover, in studies that show ZIP’s ability to reverse late-LTP maintenance, ZIP has no effect on basal synaptic transmission recorded in slices [31, 32] or in vivo [24, 33, 34], or on early-LTP maintenance [32]. Likewise, in studies that show ZIP’s ability to erase established long-term memory, the drug has no effect on short-term memory [24] (but see [35]), or on long-term memory that was recently reactivated [36]. Long-term memories that have recently been recalled may undergo an active reconsolidation process that is sensitive to blockers of consolidation, such as protein synthesis inhibitors [37], but is resistant to ZIP [36, 38]. Once the reconsolidation period has ended, ZIP’s ability to erase memory returns. This specific effect of ZIP on PKMs/atypical PKCs, late-LTP, and long-term memory maintenance, and not on full-length conventional/novel PKC isoforms or other forms of synaptic plasticity and memory, is difficult to explain by a non-specific effect of ZIP.

In addition, ZIP’s inhibitory action on PKMζ can be distinguished from its reported non-specific effects by the use of a scrambled version of the peptide as control. Whereas ZIP blocks the synaptic potentiation induced by postsynaptic perfusion of PKMζ, scrambled ZIP applied at the same doses does not [39]. In contrast, the non-specific effects reported for ZIP, such as membrane instability and “neuronal silencing,” were also reported for scrambled ZIP applied at the same doses as ZIP [21, 22], thus documenting inappropriate use or handling of the drug in these studies. The appropriate use of scrambled ZIP as a control, which has no effects in most studies of LTP and memory [32, 40, 41], provides strong necessity and erasure evidence for the maintenance role of PKMζ or a molecule with very closely related properties.

Also excluded from Lisman’s review were many studies that used agents other than ZIP to inhibit the action of PKMζ that result in the same reversal of LTP and memory maintenance. These agents include: 1) dominant negative-PKMζ [31, 42], 2) the inhibitor chelerythrine, which selectively inhibits PKM forms at low doses [31], and 3) RNAi that suppresses the expression of PKMζ [43]. RNAi that suppresses the other atypical isoform, PKCι/λ, does not disrupt memory maintenance [43]. Thus, if the studies using ZIP, chelerythrine, dominant negative-PKMζ, and PKMζ-RNAi had not been excluded from Lisman’s review, the evidence supporting PKMζ’s central role in LTP and long-term memory maintenance might have been much stronger than for CaMKII.

## Inhibiting CaMKII and PKMζ reveals their different roles in synaptic transmission and LTP

**Fig. 1:**
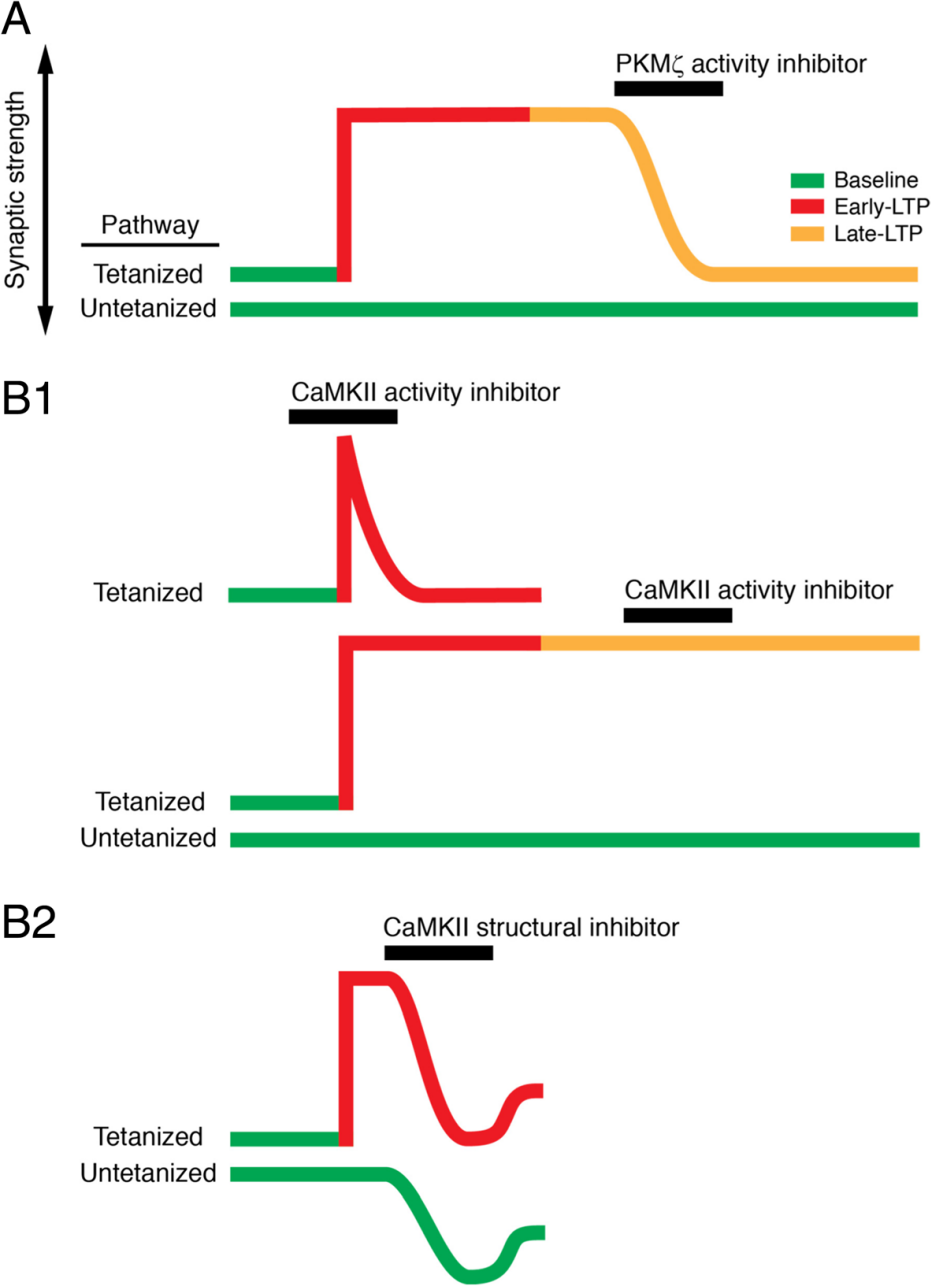
PKMζ and CaMKII inhibition differ in their effects on LTP and basal synaptic transmission. **A**) Above, schematic representation showing PKMζ inhibition reverses late-LTP when inhibitors are applied after late-LTP is established. After the inhibitor is eliminated, the potentiation does not return, indicating PKMζ’s role in maintaining late-LTP. Below, in a separate synaptic pathway, PKMζ inhibition has no effect on basal, untetanized synaptic transmission. PKMζ inhibition has no effect on early-LTP maintenance (not shown). **B**) The effects of CaMKII inhibition depend on the mechanism of inhibition. **B1**) Inhibition of CaMKII activity blocks LTP induction (above), but has no effect on LTP maintenance (middle) or basal synaptic transmission (below). **B2**) Disrupting the interaction between CaMKII and NMDAR decreases both early-LTP (above) and basal synaptic transmission (below) with no specificity for potentiated vs. unpotentiated synapses. Elimination of the blockers of CaMKII-NMDAR interaction shows incomplete reversal, indicating an effect on the maintenance of AMPAR-mediated synaptic transmission

Importantly, regardless of the method used, when the effects of inhibiting PKMζ and CaMKII are examined, the results reveal that the two molecules play very different roles in LTP and memory. All inhibitors of PKMζ, including ZIP [24, 31–34], chelerythrine [31, 44], and dominant negative mutant forms of PKM [31, 45], reverse the maintenance of late-LTP and other forms of long-term synaptic plasticity without disrupting basal synaptic transmission, recorded either in brain slices or in vivo (Fig. 1a). This ability of PKMζ inhibitors to specifically reverse late-LTP maintenance but not basal transmission or early-LTP maintenance is unique in the literature. Indeed, these experiments were the first to demonstrate that a persistently active molecular mechanism maintains late-LTP and long-term memory storage, and they remain difficult to explain by models of memory storage that are sustained solely by structurally stable synapses.

In contrast to inhibiting PKMζ, inhibiting CaMKII does not specifically reverse LTP maintenance, and the effects of the inhibition depend upon the method by which the CaMKII molecule is affected (Fig. 1b). To understand the various effects of inhibiting CaMKII, it is first important to recognize that there are two models of CaMKII’s persistent effects in LTP maintenance — the original autophosphorylation model proposed by Lisman in 1988 [8], and a structural model involving translocation of the kinase from cytosol to PSD, shown for a chemical form of LTP in 2004 [46] and formalized by Lisman in 2013 [47]. Whereas in his review Lisman conflates the evidence for the two models in support of a role for CaMKII in LTP and memory, the experiments testing the enzymatic and the structural functions of CaMKII yield very different results.

Most recent studies of the role of CaMKII’s enzymatic activity use cell-permeable versions of a peptide, CN21, that mimics the sequence of an endogenous CaMKII inhibitor, which at low doses effectively block both the Ca^2+^/CaM-stimulated and the autonomous activity of the enzyme [48]. These peptides prevent LTP when applied during the strong afferent stimulation that triggers LTP [48–50] (Fig. 1b1). But these same inhibitors when applied a few minutes after the stimulation have no effect on LTP [48–50]. The most temporally precise measurement used optical stimulation to release the inhibitory action of the peptide and showed LTP blockade when the inhibitor was activated concurrently with strong afferent synaptic stimulation, but no effect when activated ~ 1 min afterward [50]. These inhibitors applied at doses that effectively block Ca^2+^/CaM-stimulated and autonomous CaMKII activity also have no effect on basal synaptic transmission. Such a transient block of LTP, in which inhibitors prevent LTP when applied during stimulation but lose their efficacy when applied after LTP is established, is characteristic of a role in LTP induction and is identical to the effects of inhibiting many other signaling molecules that have been implicated in LTP induction [51]. As such, it is strong evidence for the role of CaMKII phosphotransferase activity in LTP induction, but not LTP maintenance.

In addition to its phosphotransferase activity, however, the abundance of CaMKIIα in the PSD (~ 80 holoenzymes associated with a typical PSD [52]) suggests that CaMKII may also have a structural role relevant to postsynaptic function. Consistent with this notion, NMDAR activation stimulates both CaMKII autophosphorylation and its translocation from cytosol to the PSD [6, 53, 54], and it has been suggested that the increased abundance of CaMKII in the PSD may help form physical “slots” for AMPARs [47]. Moreover, the same C21-based inhibitory peptides that block the enzyme’s activity at low doses disrupt CaMKII’s interaction with NMDARs in the PSD at higher doses. At these higher doses CN21 disrupts both basal AMPAR-mediated synaptic transmission and potentiated synaptic transmission during early-LTP [55–57] (Fig. 1b2). The effects of the peptide on potentiated and non-potentiated AMPAR responses are equivalent [55–57]. Thus, in contrast to PKMζ, CaMKII’s putative structural role has no specificity with respect to maintaining potentiated vs. unpotentiated synapses. Lisman speculates that a chain of molecules extend from the NMDAR-CaMKII complex to delta-catenin, to AMPA-binding protein, and then to AMPARs [47]. When the structure-disrupting peptide is washed-out, the depressed synaptic response does not fully return to the baseline response (Fig. 1b2), an effect that Lisman views as crucial evidence for a mnemonic role of CaMKII, but is equally, and perhaps more parsimoniously consistent with a role for CaMKII in maintaining postsynaptic structure.

## The disruption of synaptic transmission by CaMKII inhibition influences the interpretation of CaMKII memory “erasure” experiments

Because PKMζ inhibitors specifically reverse late-LTP maintenance and do not impair baseline synaptic transmission or early-LTP, PKMζ, or a molecule with very similar properties, is clearly crucial for maintaining late-LTP maintenance. Thus, the selective reversal of only potentiated synapses and not unpotentiated synapses induced by PKMζ inhibitors, as shown both in slices and in vivo, established a paradigm for causally linking LTP maintenance to memory storage by “erasure” experiments [24].

In contrast, CaMKII structural blockers irreversibly depress basal synaptic transmission and early-LTP maintenance equivalently [55–57]. Therefore, LTP maintenance reversal experiments that rely on CaMKII structural blockade are difficult to interpret because the inhibitors confound the possible role of CaMKII in maintaining LTP and its demonstrable role in maintaining synapse structure and general synaptic transmission. Thus, CaMKII’s role in LTP maintenance is unclear.

Analogously, Lisman’s recent memory erasure experiments involving overexpression of a dominant negative, K42-mutated form of CaMKII do not provide strong evidence for CaMKII’s role in memory maintenance [58]. In the context of endogenous CaMKII knockdown in neurons that causes a ~ 60% decrease in basal synaptic transmission, overexpression of wild-type CaMKIIα can support basal synaptic transmission, rescuing postsynaptic AMPAR transmission to normal levels [59]. In contrast, K42-mutants of CaMKIIα cannot support basal synaptic transmission [59]. Likewise, overexpression in neurons of K42-mutated CaMKIIα acts as a dominant negative mutation by inducing a general reduction in synaptic transmission by nearly 50% [60], similar to the reduction of strength of both potentiated and unpotentiated synapses observed with applications of the CaMKII-NMDAR blocking peptide. Such a non-specific reduction of synaptic transmission is likely to disrupt the function of pre-existing functional networks of neurons that are thought to maintain long-term memories. The non-specific reduction should prevent the expression of memory that was previously stored in the functional network, potentially without affecting the capacity of the newly reconfigured network to acquire new memories. The same pattern of impaired memory and spared learning in water maze tasks has been observed after temporary inactivation or permanent lesion of 40% of hippocampus [61, 62]. Thus, similar to the observations of depressed synaptic transmission in hippocampal neurons after K42-mutated CaMKIIα overexpression, the effects of K42-mutated CaMKIIα overexpression in behaving animals are most parsimoniously attributed to structural impairment of normal synaptic transmission in hippocampal circuitry rather than to a specific impairment of memory maintenance.

## Overexpression of CaMKII mutants and PKMζ

As another criterion, Lisman asserts that overexpression of maintenance molecules predicts saturation of synaptic transmission and loss of memory, but this is not necessarily the case. First, in contrast to early-LTP, which shows no further enhancement with repeated tetanization (i.e., saturation), late-LTP, the putative storage mechanism of long-term memory, does not saturate with repeated tetanization [63]. Indeed, saturation of LTP in awake rats fails to impair acquisition and recall of water maze memory, if the rats first learn the water maze task, explicitly demonstrating Lisman’s assertion is not necessarily the case [64].

In this light, the precise nature of the overexpressed kinase and the method of overexpression must be carefully considered when interpreting overexpression experiments. In contrast to postsynaptic perfusion of PKMζ that is sufficient to mimic and occlude LTP [25, 30, 31], viral overexpression of PKMζ in neurons enhances only a subset of synapses [65, 66], and most of the overexpressed PKMζ appears to be excluded from synaptic sites [67]. Additional NMDAR stimulation of the synapses allows overexpressed PKMζ to translocate to the PSD [67], in a process possibly related to PKMζ’s role as a “plasticity-related protein” (PRP) [32] during synaptic tagging [68]. This "capture" of PRPs by activated (“tagged”) synapses may be the mechanism by which viral overexpression of PKMζ enhances long-term memory, which has been observed in several tasks [42, 69, 70].

Likewise, overexpression of wild-type CaMKIIα or mutated, pseudo-T286-phosphorylated forms of CaMKIIα (T286D mutations) does not cause synaptic potentiation [71, 72]. This is because T286 is not the only site on CaMKII that undergoes autophosphorylation after Ca^2+^/CaM stimulation. T305 and T306 also autophosphorylate, but their effects are to inhibit CaMKII activity [72, 73]. Only overexpression of T305A/T306A mutations, such as CaMKIIα T286D/T305A/T306A that both mimics T286 autophosphorylation and prevents the reduction in activity by T305/T306 autophosphorylation, causes synaptic potentiation [59, 72].

Lisman’s recent occlusion experiments used overexpression of this mutant T286D/T305A/T306A version of CaMKIIα, engineered to simulate persistent activation of the kinase that is also immune to deactivation [58]. This overexpression caused amnesia [58], but what it means is unclear. This is because, although overexpression of T286D/T305A/T306A mutated CaMKIIα may simulate persistent activation of the kinase, inhibition of the kinase’s activity does not impair the maintenance of either LTP, as discussed above, or long-term memory [48, 50], making it unlikely that CaMKII activation actually persists in LTP and long-term memory.

Indeed, the overexpression within neurons of CaMKIIα containing the T305A/T306A mutations without the T286D mutation causes synaptic potentiation that is rapidly reversed by acute applications of NMDAR antagonists [59]. These findings suggest that once T286 of CaMKII undergoes autophosphorylation within neurons, the persistence of this autophosphorylation is not maintained without continual Ca^2+^/CaM stimulation [59]. Because neither LTP maintenance nor long-term memory storage is reversed by acute applications of NMDAR antagonists [74], the persistent synaptic potentiation by CaMKII overexpression and the physiological processes of LTP and memory appear to be mechanistically distinct.

## Are the actions of PKMζ and CaMKII in LTP and memory persistent?

If occlusion experiments are difficult to interpret, and the effects of CaMKII structural inhibitors on LTP and memory are confounded by their general disruption of synaptic transmission, how might one further evaluate PKMζ and CaMKII as memory maintenance molecules? An additional criterion for a maintenance mechanism might be helpful — *persistence*.

Persistence is the hallmark of PKMζ’s action during LTP maintenance — the increase in the autonomously active kinase lasts for hours in hippocampal slices [12, 25]. Recently, persistent increases in PKMζ have been examined in various forms of long-term memory. In the hippocampus, these experience-induced increases last for at least a week following trace conditioning [43] and for at least a month following spatial conditioning [75]. In neocortex, the persistence of PKMζ increases can vary in different layers and regions, lasting for at least 40 days in the output layer 5 of motor cortex during the long-term storage of skilled motor memory [38].

In contrast to the persistent increase of PKMζ in LTP, the increase in the autonomous activity of CaMKII in LTP is transient, lasting at most a few minutes after the afferent synaptic tetanization that induces LTP, as measured by either biochemical assays of endogenous CaMKII [76] or by changes in the signal of a fluorescent version of CaMKII [77]. This brief duration of CaMKII activation after stimulation is in line with the ~ 1 min time-window of the efficacy of CaMKII inhibitors, as discussed above [48, 50].

In contrast to the very short-lived increase of autonomous CaMKII activity, CaMKII translocation to the PSD has been reported to last ~ 1 h following chemically induced LTP [46]. This time course is consistent with a role in early-LTP, and thus, further work is required to examine whether the translocation persists into late-LTP maintenance or occurs during long-term memory storage in vivo.

## Do the PKMζ and CaMKII models integrate the actions of other molecules implicated in late-LTP and long-term memory?

Lastly, one might also consider whether the models for the persistent action of PKMζ and CaMKII are integrated with the many other signaling molecules implicated in LTP. Do the models help to explain and even predict experimental data on the molecular mechanisms of all phases of LTP: *induction*, *expression*, and *maintenance*?

LTP *induction* is triggered by Ca^2+^ influx through activated NMDARs, which then stimulates multiple signal transduction pathways. The persistent increase of PKMζ protein through the upregulation of PKMζ mRNA translation in LTP requires many of these transient, early signaling events, including NMDAR, CaMKII, ERK, PKA, and mTOR activation, BDNF signaling, and actin filament formation [11, 78–80]. In contrast, CaMKII autophosphorylation requires only increased intracellular Ca^2+^ through the activated NMDAR to bind to CaM [8]. CaMKII translocation from cytosol to PSD is also triggered by NMDAR activation [46], but it is not known whether other downstream signaling mechanisms including new protein synthesis are required. Furthermore, it is unclear precisely how CaMKII-NMDAR structural associations lead to increased AMPAR-mediated synaptic transmission.

The mechanism of *expression* by which PKMζ enhances postsynaptic AMPAR responses during late-LTP is through stabilizing GluA2-containing AMPARs and decreasing GluA2-mediated endocytosis [26, 29, 66]. These findings led to the discovery that agents that destabilize postsynaptic GluA2 can also disrupt established late-LTP and long-term memory [29, 81], and, conversely, that inhibitors of AMPAR endocytosis not only block the amnestic effects of ZIP [26], but also, when applied by themselves, prolong LTP and long-term memory maintenance [82–84]. Further work will be required to see if the models of CaMKII persistence have equivalent explanatory and predictive powers.

Finally, as discussed above, late-LTP *maintenance* is reversed by all known PKMζ inhibitors, and early-LTP and basal synaptic transmission are not affected by these agents. This specific action on late-LTP maintenance and not early-LTP or basal synaptic transmission is not explained by the CaMKII autophosphorylation or structural models. Conversely, however, the ability of CaMKII activity inhibitors to suppress LTP induction can be explained by observations that CaMKII activity is required to induce new PKMζ synthesis [78]. Likewise, CaMKII’s structural role may be important in maintaining the postsynaptic neurotransmission that PKMζ modulates in late-LTP.

## Conclusions

When the criteria of *necessity*, *occlusion*, *erasure*, and *persistence* are examined in detail, the cumulative evidence strongly supports the persistent action of PKMζ as a core molecular mechanism of late-LTP and long-term memory maintenance. CaMKII appears to have two roles: an enzymatic role that is essential for the induction of LTP, and a structural role involving interaction with the NMDAR that maintains synaptic transmission regardless of the state of potentiation. Further work will be required to evaluate whether this structural role of CaMKII is also: 1) one of several transient, post-translational mechanisms upregulated in early-LTP, 2) an expression mechanism of late-LTP and long-term memory, downstream of maintenance by PKMζ, or 3) as Lisman proposed, a maintenance mechanism of a form of synaptic plasticity, independent of PKMζ. A fundamental difference between the molecules is that CaMKII structural inhibitors generally disrupt AMPAR-mediated synaptic transmission, including basal transmission, whereas PKMζ inhibitors specifically disrupt only potentiated synaptic transmission during late-LTP. Thus, one scenario that remains to be fully investigated is that CaMKII maintains the synaptic plasticity involving the initial “AMPAfication” of NMDAR-only, “silent” synapses that occurs during development, and PKMζ maintains further potentiation of only a few of these NMDAR/AMPAR-containing synapses to sparsely encode and store information acquired during learning and experience. With John gone, we hope someone picks up his mantle to explore these and other possibilities.

## References

[1] Kandel ER, Schwartz JH (1982). Molecular biology of learning: modulation of transmitter release. Science.

[2] Kandel ER (2001). The molecular biology of memory storage: a dialogue between genes and synapses. Science.

[3] Schwartz JH (1993). Cognitive kinases. Proc Natl Acad Sci U S A.

[4] Crick F (1984). Memory and molecular turnover. Nature.

[5] Schwartz JH, Greenberg SM (1987). Molecular mechanisms for memory: second-messenger induced modifications of protein kinases in nerve cells. Annu Rev Neurosci.

[6] Saitoh T, Schwartz JH (1985). Phosphorylation-dependent subcellular translocation of a Ca2+/calmodulin-dependent protein kinase produces an autonomous enzyme in *Aplysia* neurons. J Cell Biol.

[7] Miller SG, Kennedy M (1986). Regulation of brain type II Ca2+/calmodulin-dependent protein kinase by autophosphorylation: a Ca2+−triggered molecular switch. Cell.

[8] Lisman JE, Goldring MA (1988). Feasibility of long-term storage of graded information by the Ca2+/calmodulin-dependent protein kinase molecules of the postsynaptic density. Proc Natl Acad Sci U S A.

[9] Lisman JE. A mechanism for memory storage insensitive to molecular turnover: a bistable autophosphorylating kinase. Proc Natl Acad Sci USA. 1985;82:3055-57. 10.1073/pnas.82.9.3055 PMC397705 2986148

[10] Nishizuka Y (1995). Protein kinase C and lipid signaling for sustained cellular responses. FASEB J.

[11] Sacktor TC, Osten P, Valsamis H, Jiang X, Naik MU, Sublette E (1993). Persistent activation of the ζ isoform of protein kinase C in the maintenance of long-term potentiation. Proc Natl Acad Sci U S A.

[12] Osten P, Valsamis L, Harris A, Sacktor TC. Protein synthesis-dependent formation of protein kinase Mζ in long-term potentiation. J Neurosci. 1996;16:2444-51. 10.1523/JNEUROSCI.16-08-02444.1996 PMC6578779 8786421

[13] Hernandez AI, Blace N, Crary JF, Serrano PA, Leitges M, Libien JM, Weinstein G, Tcherapanov A, Sacktor TC. Protein kinase Mζ synthesis from a brain mRNA encoding an independent protein kinase Cζ catalytic domain. Implications for the molecular mechanism of memory. J Biol Chem. 2003;278:40305-16. 10.1074/jbc.M307065200 12857744

[14] Muslimov IA, Nimmrich V, Hernandez AI, Tcherepanov A, Sacktor TC, Tiedge H (2004). Dendritic transport and localization of protein kinase Mζ mRNA: implications for molecular memory consolidation. J Biol Chem..

[15] Lisman J, Malenka RC, Nicoll RA, Malinow R. Learning mechanisms: the case for CaM-KII. Science. 1997;276:2001–2. 10.1126/science.276.5321.2001 9221509

[16] Lisman J. Criteria for identifying the molecular basis of the engram (CaMKII, PKMzeta). Molecular Brain. 2017;10:55. 10.1186/s13041-017-0337-4 PMC5707903 29187215

[17] Sacktor TC, Hell JW. The genetics of PKMzeta and memory maintenance. Sci Signal. 2017;10:eaao2327. 10.1126/scisignal.aao2327 PMC6171341 29138296

[18] Morris RG. Forget me not. eLife. 2016;5:e16597. 10.7554/eLife.16597 PMC4869910 27187147

[19] Lee AM, Kanter BR, Wang D, Lim JP, Zou ME, Qiu C, McMahon T, Dadgar J, Fischbach-Weiss SC, Messing RO (2013). Prkcz null mice show normal learning and memory. Nature.

[20] Volk LJ, Bachman JL, Johnson R, Yu Y, Huganir RL (2013). PKM-zeta is not required for hippocampal synaptic plasticity, learning and memory. Nature.

[21] Sadeh N, Verbitsky S, Dudai Y, Segal M. Zeta inhibitory peptide, a candidate inhibitor of protein kinase Mzeta, is excitotoxic to cultured hippocampal neurons. J Neurosci. 2015;35:12404–11. 10.1523/JNEUROSCI.0976-15.2015 PMC6605392 26354909

[22] LeBlancq MJ, McKinney TL, Dickson CT (2016). ZIP it: neural silencing is an additional effect of the PKM-zeta inhibitor zeta-inhibitory peptide. J Neurosci.

[23] Barry JM, Rivard B, Fox SE, Fenton AA, Sacktor TC, Muller RU (2012). Inhibition of protein kinase Mzeta disrupts the stable spatial discharge of hippocampal place cells in a familiar environment. J Neurosci.

[24] Pastalkova E, Serrano P, Pinkhasova D, Wallace E, Fenton AA, Sacktor TC (2006). Storage of spatial information by the maintenance mechanism of LTP. Science.

[25] Tsokas P, Hsieh C, Yao Y, Lesburgueres E, Wallace EJ, Tcherepanov A, Jothianandan D, Hartley BR, Pan L, Rivard B, et al. Compensation for PKMzeta in long-term potentiation and spatial long-term memory in mutant mice. eLife. 2016;5:e14846. 10.7554/eLife.14846 PMC4869915 27187150

[26] Migues PV, Hardt O, Wu DC, Gamache K, Sacktor TC, Wang YT, Nader K (2010). PKMζ maintains memories by regulating GluR2-dependent AMPA receptor trafficking. Nat Neurosci.

[27] Li YQ, Xue YX, He YY, Li FQ, Xue LF, Xu CM, Sacktor TC, Shaham Y, Lu L (2011). Inhibition of PKMzeta in nucleus accumbens core abolishes long-term drug reward memory. J Neurosci.

[28] Pauli WM, Clark AD, Guenther HJ, O'Reilly RC, Rudy JW (2012). Inhibiting PKMzeta reveals dorsal lateral and dorsal medial striatum store the different memories needed to support adaptive behavior. Learn Mem.

[29] Yao Y, Kelly MT, Sajikumar S, Serrano P, Tian D, Bergold PJ, Frey JU, Sacktor TC (2008). PKMζ maintains late long-term potentiation by N-ethylmaleimide-sensitive factor/GluR2-dependent trafficking of postsynaptic AMPA receptors. J Neurosci.

[30] Ling DS, Benardo LS, Sacktor TC (2006). Protein kinase Mζ enhances excitatory synaptic transmission by increasing the number of active postsynaptic AMPA receptors. Hippocampus.

[31] Ling DS, Benardo LS, Serrano PA, Blace N, Kelly MT, Crary JF, Sacktor TC (2002). Protein kinase Mζ is necessary and sufficient for LTP maintenance. Nat Neurosci.

[32] Sajikumar S, Navakkode S, Sacktor TC, Frey JU (2005). Synaptic tagging and cross-tagging: the role of protein kinase Mζ in maintaining long-term potentiation but not long-term depression. J Neurosci.

[33] Madronal N, Gruart A, Sacktor TC, Delgado-Garcia JM (2010). PKMζ inhibition reverses learning-induced increases in hippocampal synaptic strength and memory during trace eyeblink conditioning. PLoS One.

[34] Cooke SF, Bear MF (2010). Visual experience induces long-term potentiation in the primary visual cortex. J Neurosci.

[35] Evuarherhe O, Barker GR, Savalli G, Warburton EC, Brown MW (2014). Early memory formation disrupted by atypical PKC inhibitor ZIP in the medial prefrontal cortex but not hippocampus. Hippocampus.

[36] Levitan D, Fortis-Santiago Y, Figueroa JA, Reid EE, Yoshida T, Barry NC, Russo A, Katz DB (2016). Memory retrieval has a dynamic influence on the maintenance mechanisms that are sensitive to zeta-inhibitory peptide (ZIP). J Neurosci.

[37] Nader K, Schafe GE, LeDoux JE (2000). The labile nature of consolidation theory. Nat Rev Neurosci.

[38] Gao PP, Goodman JH, Sacktor TC, Francis JT (2018). Persistent increases of PKMζ in sensorimotor cortex maintain procedural long-term memory storage. iScience.

[39] Yao Y, Shao C, Jothianandan D, Tcherepanov A, Shouval H, Sacktor TC (2013). Matching biochemical and functional efficacies confirm ZIP as a potent competitive inhibitor of PKMzeta in neurons. Neuropharmacology.

[40] Serrano P, Yao Y, Sacktor TC (2005). Persistent phosphorylation by protein kinase Mζ maintains late-phase long-term potentiation. J Neurosci.

[41] Serrano P, Friedman EL, Kenney J, Taubenfeld SM, Zimmerman JM, Hanna J, Alberini C, Kelley AE, Maren S, Rudy JW (2008). PKMζ maintains spatial, instrumental, and classically conditioned long-term memories. PLoS Biol.

[42] Shema R, Haramati S, Ron S, Hazvi S, Chen A, Sacktor TC, Dudai Y (2011). Enhancement of consolidated long-term memory by overexpression of protein kinase Mzeta in the neocortex. Science.

[43] Wang S, Sheng T, Ren S, Tian T, Lu W. Distinct roles of PKCiota/lambda and PKMzeta in the initiation and maintenance of hippocampal long-term potentiation and memory. Cell Rep. 2016;16:1954–61. 10.1016/j.celrep.2016.07.030 27498875

[44] Cai D, Pearce K, Chen S, Glanzman DL (2011). Protein kinase M maintains long-term sensitization and long-term facilitation in Aplysia. J Neurosci.

[45] Hu J, Adler K, Farah CA, Hastings MH, Sossin WS, Schacher S (2017). Cell-specific PKM isoforms contribute to the maintenance of different forms of persistent long-term synaptic plasticity. J Neurosci.

[46] Otmakhov N, Tao-Cheng JH, Carpenter S, Asrican B, Dosemeci A, Reese TS, Lisman J (2004). Persistent accumulation of calcium/calmodulin-dependent protein kinase II in dendritic spines after induction of NMDA receptor-dependent chemical long-term potentiation. J Neurosci.

[47] Sanhueza M, Lisman J. The CaMKII/NMDAR complex as a molecular memory. Molecular Brain. 2013;6:10. 10.1186/1756-6606-6-10 PMC3582596 23410178

[48] Buard I, Coultrap SJ, Freund RK, Lee YS, Dell'Acqua ML, Silva AJ, Bayer KU (2010). CaMKII “autonomy” is required for initiating but not for maintaining neuronal long-term information storage. J Neurosci.

[49] Otmakhov N, Griffith LC, Lisman JE. Postsynaptic inhibitors of calcium/calmodulin-dependent protein kinase type II block induction but not maintenance of pairing-induced long-term potentiation. J Neurosci. 1997;17:5357–65. 10.1523/JNEUROSCI.17-14-05357.1997 PMC6793827 9204920

[50] Murakoshi H, Shin ME, Parra-Bueno P, Szatmari EM, Shibata AC, Yasuda R. Kinetics of endogenous CaMKII required for synaptic plasticity revealed by optogenetic kinase inhibitor. Neuron. 2017;94:37–47. 10.1016/j.neuron.2017.02.036 PMC5425291 28318784

[51] Sanes JR, Lichtman JW (1999). Can molecules explain long-term potentiation?. Nat Neurosci.

[52] Chen X, Vinade L, Leapman RD, Petersen JD, Nakagawa T, Phillips TM, Sheng M, Reese TS (2005). Mass of the postsynaptic density and enumeration of three key molecules. Proc Natl Acad Sci U S A.

[53] Shen K, Meyer T (1999). Dynamic control of CaMKII translocation and localization in hippocampal neurons by NMDA receptor stimulation. Science.

[54] Bayer KU, LeBel E, McDonald GL, O'Leary H, Schulman H, De Koninck P (2006). Transition from reversible to persistent binding of CaMKII to postsynaptic sites and NR2B. J Neurosci.

[55] Sanhueza M, McIntyre CC, Lisman JE (2007). Reversal of synaptic memory by Ca2+/calmodulin-dependent protein kinase II inhibitor. J Neurosci.

[56] Sanhueza M, Fernandez-Villalobos G, Stein IS, Kasumova G, Zhang P, Bayer KU, Otmakhov N, Hell JW, Lisman J (2011). Role of the CaMKII/NMDA receptor complex in the maintenance of synaptic strength. J Neurosci.

[57] Barcomb K, Hell JW, Benke TA, Bayer KU (2016). The CaMKII/GluN2B protein interaction maintains synaptic strength. J Biol Chem.

[58] Rossetti T, Banerjee S, Kim C, Leubner M, Lamar C, Gupta P, Lee B, Neve R, Lisman J. Memory erasure experiments indicate a critical role of CaMKII in memory storage. Neuron. 2017;96:207–16. 10.1016/j.neuron.2017.09.010 PMC5634137 28957669

[59] Incontro S, Diaz-Alonso J, Iafrati J, Vieira M, Asensio CS, Sohal VS, Roche KW, Bender KJ, Nicoll RA (2018). The CaMKII/NMDA receptor complex controls hippocampal synaptic transmission by kinase-dependent and independent mechanisms. Nat Commun.

[60] Kabakov AY, Lisman JE. Catalytically dead αCaMKII K42M mutant acts as a dominant negative in the control of synaptic strength. PLoS One. 2015;10:e0123718. 10.1371/journal.pone.0123718 PMC4408036 25905720

[61] Moser EI, Krobert KA, Moser MB, Morris RG (1998). Impaired spatial learning after saturation of long-term potentiation. Science.

[62] Kubik S, Fenton AA (2005). Behavioral evidence that segregation and representation are dissociable hippocampal functions. J Neurosci.

[63] Frey U, Schollmeier K, Reymann KG, Seidenbecher T (1995). Asymptotic hippocampal long-term potentiation in rats does not preclude additional potentiation at later phases. Neuroscience.

[64] Otnaess MK, Brun VH, Moser MB, Moser EI (1999). Pretraining prevents spatial learning impairment after saturation of hippocampal long-term potentiation. J Neurosci.

[65] Ron S, Dudai Y, Segal M. Overexpression of PKMzeta alters morphology and function of dendritic spines in cultured cortical neurons. Cereb Cortex. 2012;22:2519–28. 10.1093/cercor/bhr323 PMC4705334 22123937

[66] Yu NK, Uhm H, Shim J, Choi JH, Bae S, Sacktor TC, Hohng S, Kaang BK. Increased PKMzeta activity impedes lateral movement of GluA2-containing AMPA receptors. Molecular Brain. 2017;10:56. 10.1186/s13041-017-0334-7 PMC5716381 29202853

[67] Palida SF, Butko MT, Ngo JT, Mackey MR, Gross LA, Ellisman MH, Tsien RY (2015). PKMzeta, but not PKClambda, is rapidly synthesized and degraded at the neuronal synapse. J Neurosci.

[68] Frey U, Morris RG (1997). Synaptic tagging and long-term potentiation. Nature.

[69] Schuette SR, Fernandez-Fernandez D, Lamla T, Rosenbrock H, Hobson S. Overexpression of protein kinase Mzeta in the hippocampus enhances long-term potentiation and long-term contextual but not cued fear memory in rats. J Neurosci. 2016;36:4313–24. 10.1523/JNEUROSCI.3600-15.2016 PMC4829652 27076427

[70] Yan W, Liu JF, Han Y, Zhang W, Luo YX, Xue YX, Zhu WL, Yang C, Chen WH, Guo HL, et al. Protein kinase Mzeta in medial prefrontal cortex mediates depressive-like behavior and antidepressant response. Mol Psychiatry. 2018;23:1–14. 10.1038/mp.2017.219 29180675

[71] Mayford M, Wang J, Kandel E, O'Dell T (1995). CaMKII regulates the frequency-response function of hippocampal synapses for the production of both LTD and LTP. Cell.

[72] Pi HJ, Otmakhov N, Lemelin D, De Koninck P, Lisman J (2010). Autonomous CaMKII can promote either long-term potentiation or long-term depression, depending on the state of T305/T306 phosphorylation. J Neurosci.

[73] Lou LL, Schulman H (1989). Distinct autophosphorylation sites sequentially produce autonomy and inhibition of the multifunctional Ca2+/calmodulin-dependent protein kinase. J Neurosci.

[74] Morris RG. NMDA receptors and memory encoding. Neuropharmacology. 2013;74:32–40. 10.1016/j.neuropharm.2013.04.014 23628345

[75] Hsieh C, Tsokas P, Serrano P, Hernandez AI, Tian D, Cottrell JE, Shouval HZ, Fenton AA, Sacktor TC (2017). Persistent increased PKMzeta in long-term and remote spatial memory. Neurobiol Learn Mem.

[76] Lengyel I, Voss K, Cammarota M, Bradshaw K, Brent V, Murphy KP, Giese KP, Rostas JA, Bliss TV (2004). Autonomous activity of CaMKII is only transiently increased following the induction of long-term potentiation in the rat hippocampus. Eur J Neurosci.

[77] Lee SJ, Escobedo-Lozoya Y, Szatmari EM, Yasuda R (2009). Activation of CaMKII in single dendritic spines during long-term potentiation. Nature.

[78] Kelly MT, Crary JF, Sacktor TC (2007). Regulation of protein kinase Mζ synthesis by multiple kinases in long-term potentiation. J Neurosci.

[79] Kelly MT, Yao Y, Sondhi R, Sacktor TC (2006). Actin polymerization regulates the synthesis of PKMζ in LTP. Neuropharmacology.

[80] Mei F, Nagappan G, Ke Y, Sacktor TC, Lu B (2011). BDNF facilitates L-LTP maintenance in the absence of protein synthesis through PKMzeta. PLoS One.

[81] Migues PV, Hardt O, Finnie P, Wang YW, Nader K. The maintenance of long-term memory in the hippocampus depends on the interaction between N-ethylmaleimide-sensitive factor and GluA2. Hippocampus. 2014;24:1112–9. 10.1002/hipo.22295 24753224

[82] Dong Z, Han H, Li H, Bai Y, Wang W, Tu M, Peng Y, Zhou L, He W, Wu X (2015). Long-term potentiation decay and memory loss are mediated by AMPAR endocytosis. J Clin Invest.

[83] Migues PV, Liu L, Archbold GE, Einarsson EO, Wong J, Bonasia K, Ko SH, Wang YT, Hardt O (2016). Blocking synaptic removal of GluA2-containing AMPA receptors prevents the natural forgetting of long-term memories. J Neurosci.

[84] Awasthi A, Ramachandran B, Ahmed S, Benito E, Shinoda Y, Nitzan N, Heukamp A, Rannio S, Martens H, Barth J, et al., Synaptotagmin-3 drives AMPA receptor endocytosis, depression of synapse strength, and forgetting. Science. 2018; 10.1126/science.aav1483. 10.1126/science.aav1483 30545844

